# Exploiting the reference genome sequence of hexaploid wheat: a proteomic study of flour proteins from the cultivar Chinese Spring

**DOI:** 10.1007/s10142-019-00694-z

**Published:** 2019-06-27

**Authors:** Susan B. Altenbach, Han-Chang Chang, Annamaria Simon-Buss, Toni Mohr, Naxin Huo, Yong Q. Gu

**Affiliations:** USDA-ARS Western Regional Research Center, 800 Buchanan Street, Albany, CA 94710 USA

**Keywords:** Gluten proteins, Gliadins, Low-molecular weight glutenin subunits, 2-DE, MS/MS, Immunogenic potential, Flour quality

## Abstract

Although the economic value of wheat flour is determined by the complement of gluten proteins, these proteins have been challenging to study because of the complexity of the major protein groups and the tremendous sequence diversity among wheat cultivars. The completion of a high-quality wheat genome sequence from the reference wheat Chinese Spring recently facilitated the assembly and annotation of a complete set of gluten protein genes from a single cultivar, making it possible to link individual proteins in the flour to specific gene sequences. In a proteomic analysis of total wheat flour protein from Chinese Spring using quantitative two-dimensional gel electrophoresis combined with tandem mass spectrometry, gliadins or low-molecular-weight glutenin subunits were identified as the predominant proteins in 72 protein spots. Individual spots were associated with 40 of 56 Chinese Spring gene sequences, including 16 of 26 alpha gliadins, 10 of 11 gamma gliadins, six of seven omega gliadins, one of two delta gliadins, and nine of ten LMW-GS. Most genes that were not associated with protein spots were either expressed at low levels in endosperm or encoded proteins with high similarity to other proteins. A wide range of protein accumulation levels were observed and discrepancies between transcript levels and protein levels were noted. This work together with similar studies using other commercial cultivars should provide new insight into the molecular basis of wheat flour quality and allergenic potential.

**Electronic supplementary material:**

The online version of this article (10.1007/s10142-019-00694-z) contains supplementary material, which is available to authorized users.

## Introduction

Gluten proteins comprise about 70% of the total protein in wheat flour and are largely responsible for the functional properties that make it possible to produce a wide range of different food products from flour. However, despite many years of study, the large numbers and closely related repetitive structures of these proline- and glutamine-rich proteins coupled with their sequence diversity in different cultivars have made it particularly challenging to relate specific proteins to flour end-use quality. Some of the gluten proteins also trigger human health conditions, including celiac disease, food allergies, and non-celiac wheat sensitivities and very similar proteins often differ in immunogenic potential. Gluten proteins consist of two types of proteins, the monomeric gliadins that confer extensibility to wheat flour dough and the polymeric glutenins that confer elasticity to wheat flour dough (Shewry et al. 2003, for review). The gliadins are divided into four complex groups, termed alpha, gamma, delta, and omega gliadins, each containing numerous members with similar structures and distinct repetitive motifs. Most alpha gliadins contain six cysteine residues while most gamma and delta gliadins contain eight cysteines all of which form intramolecular disulfide bonds. In comparison, most omega gliadins consist entirely of repetitive sequences and do not contain any cysteine. The glutenins consist of high-molecular-weight glutenin subunits (HMW-GS) and low-molecular-weight glutenin subunits (LMW-GS) that are linked via intermolecular disulfide bonds to form large polymers that are essential for flour end-use quality. A number of proteins with sequences similar to alpha, gamma, and omega gliadins contain an odd number of cysteine residues and are also linked into the glutenin polymer. It has been hypothesized that these proteins, referred to as chain terminators, limit the size of the glutenin polymer and have a negative effect on end-use quality (Tao and Kasarda 1989).

The gluten proteins are encoded at several different locations in the hexaploid wheat genome. HMW-GS are encoded at the *Glu-1* loci on the long arms of the group 1 homoeologous chromosomes while LMW-GS, gamma, omega, and delta gliadins are clustered at the *Glu-3* and *Gli-1* loci on the short arms of the same chromosomes. Alpha gliadins are encoded at the *Gli-2* loci located on the short arms of the group 6 chromosomes. It was originally estimated from hybridization analyses that a single hexaploid wheat cultivar could have six HMW-GS genes, more than 20 LMW-GS genes, as many as 30 gamma gliadin genes, and up to 150 alpha gliadin genes (Anderson et al. 1997; Forde et al. 1985; Sabelli and Shewry 1991). However, at least some of these genes are pseudogenes that are not expressed. Genomic sequencing data now suggest that the complexity of the gluten protein gene families, especially the alpha gliadins, may be less than initially reported. Recently, as a result of the International Wheat Genome Sequencing Consortium efforts, a high-quality genome sequence of the reference wheat cultivar Chinese Spring was published that was based on Illumina short reads (IWGC 2018; Zimin et al. 2017). However, many gluten protein genes identified in this dataset contained gaps, some of which were more than 1 kb in length, particularly in the repetitive regions (Juhász et al. 2018). Other genes were mis-assembled or were only gene fragments. Because PacBio long sequencing reads can span the entire gluten gene region without gaps, Huo et al. (2018a, b) also used PacBio assembly data along with BioNano genome maps to reconstruct the genomic regions for the gluten protein genes. They assembled and annotated a complete set of gliadin and LMW-GS genes that included 47 alpha gliadin, 14 gamma gliadin, 5 delta gliadin, 19 omega gliadin, and 17 LMW-GS genes. Of these, genes for 26 alpha gliadins, 11 gamma gliadins, two delta gliadins, five omega gliadins, and 10 LMW-GS encoded full-length proteins while the remaining genes were either partial gene sequences or pseudogenes containing premature stop codons or frameshift mutations. Sequences were further validated by mapping Illumina transcriptome reads and manually checking the alignments.

It is important to assess the full complement and accumulation levels of gluten proteins in flour from wheat cultivated throughout the world to develop a better understanding of how allelic variations and environmental factors influence the functional properties and immunogenic potential of the flour. Such studies are also critical for assessing the precise effects of genetic modifications induced by either biotechnology or breeding approaches on flour protein composition. Proteomic methods that combine two-dimensional gel electrophoresis (2-DE) and mass spectrometry (MS) have been valuable for these studies. However, the studies are challenging, in part because the gluten proteins consist of large numbers of similar proteins with overlapping MWs and pIs that are difficult to separate. Additionally, the identification of proteins by a combined 2-DE/MS method requires that the protein sequence is represented in the database used for the analysis of spectral data. The numbers of gluten protein sequences in databases have grown significantly in recent years, but these represent allelic variants from many different cultivars and ancestral species. With the genome sequencing of Chinese Spring, a complete set of gluten protein sequences from a single cultivar now has become available.

In this study, total proteins were extracted from flour of the reference wheat Chinese Spring and separated by two-dimensional gel electrophoresis (2-DE). More than 150 protein spots were excised from gels and identified by tandem mass spectrometry (MS/MS). The amounts of individual proteins accumulated in the flour were also determined by quantitative 2-DE. The goals of the study were to determine how many proteins from Chinese Spring flour could be linked to gliadin and LMW-GS genes annotated by Huo et al. (2018a, b) and to assess how well previously reported transcript data correlates with proteomic data.

## Materials and methods

### Analysis of protein sequences

Protein sequences from the hexaploid wheat *Triticum aestivum* cv. Chinese Spring were deduced from gene sequences reported by Huo et al. (2018a, b). Signal peptide cleavage sites were determined using the SignalP 4.1 Server (http://www.cbs.dtu.dk/services/SignalP/) and additional processing of specific omega gliadins and LMW-GS by an asparaginyl endoprotease was considered according to Dupont et al. (2004). MWs and pIs of the mature proteins were predicted using ProtParam (ExPASy Bioinformatic Resource Portal, https://web.expasy.org/protparam/). The numbers of epitopes relevant for celiac disease (CD) or the serious food allergy wheat-dependent exercise-induced anaphylaxis (WDEIA) as reported by Sollid et al. (2012) and Matsuo et al. (2004), respectively, were determined manually for each protein sequence.

### Extraction of protein and quantitative analysis by 2-DE

*Triticum aestivum* cv. Chinese Spring grown in a greenhouse was kindly provided by Dr. Mingcheng Luo from UC Davis and was of the same origin as that used for generating the reference genome sequence (Zimin et al. 2017). Grain was pulverized into a fine powder and sifted through a series of mesh screens. Total proteins were extracted from the resulting flour using SDS buffer (2% SDS, 10% glycerol, 50 mM DTT, 40 mM Tris-Cl, pH 6.8) for 1 h at room temperature with intermittent mixing. Insoluble material was removed by centrifugation at 16,000 g for 10 min as described in detail in Dupont et al. (2011). Three separate protein extractions were performed. Following precipitation of proteins with acetone and determination of protein concentrations according to Lowry et al. (1951), samples were analyzed by 2-DE using capillary tube gels with a pI range of 3–10 in the first dimension and NuPAGE 4–12% Bis-Tris protein gels (Life Technologies, Carlsbad, CA) in the second dimension as described in detail in Dupont et al. (2011). Each of the three extracted samples was analyzed in triplicate. Following staining with Coomassie G-250 (Sigma-Aldrich, St. Louis, MO), the resulting gels were digitized with a calibrated scanner and analyzed using Progenesis SameSpots software, Version 5.0 (Nonlinear Dynamics, Limited, Newcastle upon Tyne, UK). Gels were aligned and spots were detected by the software. All detected spots were inspected manually and adjustments were made as necessary. Normalized spot volumes determined by the software are reported for replicates of each extraction along with average values and standard deviations. For 2-DE spots in which more than one gliadin or LMW-GS were identified, the average normalized spot volume was divided among the proteins in the spot according to the percentage of unique peptides identified by MS/MS for each component.

### MS/MS analysis of protein spots

Protein spots excised from 2-D gels were reduced, alkylated, and digested with trypsin, chymotrypsin, or thermolysin using a DigestPro (Intavis, Inc., Koeln, Germany) according to the manufacturer’s instructions and analyzed by MS/MS using an EASY-nLC II interfaced with a nano-electrospray source to an Orbitrap Elite mass spectrometer (Thermo Scientific, San Jose, CA). Data acquisition parameters were described previously by Vensel et al. (2014). After conversion of raw data files to MGF files, the data were used to interrogate a database that contained Triticeae sequences downloaded from NCBI on 06-18-2018 plus full-length Chinese Spring gluten protein sequences reported by Huo et al. (2018a, b), full-length Butte 86 sequences from Dupont et al. (2011) and Altenbach et al. (2011b) and full-length Xioayan 81 gliadin sequences from Wang et al. (2017) that were not present in NCBI at the time the database was created. Common MS contaminant sequences contained in the common Repository of Adventitious Proteins (cRAP) (ftp://ftp.thegpm.org/fasta/cRAP/crap.fasta) were also added to the database to ensure that potentially contaminating proteins in the samples were identified correctly. All duplicate sequences were filtered out of the database prior to use. The database contained 125,400 entries. Two search engines, Mascot (www.matrixscience.com) and XTandem! (https://www.thegpm.org/TANDEM/*),* were used for the analyses. Data from the two searches were compiled and further validated using Scaffold version 4.7.5 (http://www.proteomesoftware.com/). Thresholds in Scaffold were set to 99% protein probability, 4 peptides, and 95% 20 ppm peptide probability. The decoy false discovery rate (FDR) was 0%. MS data from those spots that contained gluten proteins were also searched against a database that contained only the full-length gluten protein sequences from Chinese Spring and the contaminant sequences. Thresholds in Scaffold were 99% protein probability, five peptides, and 95% 20 ppm peptide probability. The predominant protein in each spot was deemed to be the protein that was assigned the greatest number of unique peptides. In cases where other proteins had at least half the number of unique peptides as the predominant protein, those proteins are reported along with the numbers of unique peptides, total spectra and protein coverage for each.

## Results

### Characterization of gluten proteins in Chinese Spring

The availability of sequences for the entire complement of gliadin and LMW-GS genes in the reference wheat Chinese Spring makes it possible to predict the characteristics of a complex set of flour proteins that are otherwise very difficult to separate, identify, and quantify. Based on the sizes of proteins deduced from the full-length gene sequences, Chinese Spring flour would be expected to contain 26 alpha gliadins that range from 30.0 to 36.2 kDa and have pIs from 6.18 to 8.28. Sixteen of these were also reported in the dataset of Juhász et al. (2018) (Table 1). Two proteins would be expected to lie outside the bulk of the alpha gliadins on 2-D gels, alpha-B3 with a predicted size of 36.2 kDa and alpha-B8 with a pI of 8.28. Alpha gliadins contain two poly Q regions that vary in size among proteins encoded by the different family members. The poly Q I region ranges from nine residues in alpha-D9 to 36 residues in alpha-A5 while the poly Q II region ranges from six residues in alpha-A6 and -D12 to 45 residues in alpha-B3. There is also striking similarity among the sequences of many of the proteins. For example, alpha-B7 and -B9 differ by only a single amino acid and the main differences between six alpha gliadins encoded by the B genome (alpha-B11, -B14, -B15, -B16, -B17, -B18) are found in the poly Q regions (Supplementary Fig. 1). Alpha-B11 and B-14 differ by one amino acid in the poly Q II region and one other substitution while alpha-B15 and -B16 differ by six glutamines in the poly Q II region and one other amino acid substitution.

**Table 1:** Characteristics of proteins deduced from full-length alpha gliadin genes reported by Huo et al. (2018b) in Chinese Spring, comparison with proteins deduced from sequences reported by Juhász et al. (2018), protein spot numbers and accumulation levels in 2-D gels, and comparison with transcript levels reported by Huo et al. (2018b)

Gene	Predicted MW^a^	Predicted pI^a^	# cys	# Q in polyQ I	# Q in polyQ II	# CD epitopes^b^	Included in Juhász et al. (2018)	2-D spot numbers^c^	% total normalized spot volume^d^	FPKM at 20 DPA^e^
α-A1	31,440	6.37	6	14	9	1		111, 113, 114	1.4	14,391
α-A2	34,471	6.50	6	29	14	1	x	81, 82, 83, 84	0.8	8321
α-A4	30,506	7.12	6	14	7	2	x	119, 120	1.8	18,005
α-A5	33,479	6.62	6	36	8	2	x			3689
α-A6	30,621	7.79	6	16	6	2	x	121, 122, 123	0.5	6536
α-A8	31,050	7.08	6	17	7	2	x			145
α-A9	32,181	6.53	6	23	12	1	x			7666
α-A10	29,996	6.18	6	11	7	2	x	117, 118	0.8	8038
α-B3	36,206	7.76	6	22	45	1		62	0.3	6749
α-B7	33,968	7.12	6	18	23	0		85, 104	0.5	19,308
α-B8	34,781	8.28	6	23	25	0	x	86, 91	0.9	7305
α-B9	33,977	7.16	6	18	22	0	x	105	0.9	23,856
α-B11	31,535	7.07	6	17	16	0				13,506
α-B14	31,413	7.02	6	17	16	0		109, 112	0.7	8489
α-B15	31,284	7.02	6	15	23	0	x			11,725
α-B16	32,054	6.42	6	19	18	0	x	113	0.1	11,549
α-B17	32,039	6.42	6	19	16	0		82	0.2	0
α-B18	31,829	7.12	6	15	13	0	x			2659
α-B25	33,818	7.78	6	12	36	1	x			935
α-D1	30,699	6.53	6	13	12	1				132
α-D4	31,542	6.79	6	16	10	3	x	108, 109	1.4	12,960
α-D5	33,412	7.75	6	20	12	8^f^	x	103, 106, 110, 112	1.7	27,257
α-D6	31,706	6.53	7	13	14	5		102	0.4	9152
α-D8	31,435	6.53	7	14	11	6		84, 85, 107, 111	2.1	2092
								113, 114		
α-D9	30,810	7.16	6	9	8	6				2742
α-D12	30,175	6.44	6	11	6	0	x			18,651

^a^Mature protein following removal of signal peptide

^b^CD-relevant epitopes include DQ2.5-glia-α1a (PFPQPQLPY), DQ2.5-glia-α1b (PYPQPQLPY), DQ2.5-glia-α2 (PQPQLPYPQ), DQ2.5-glia-α3 (FRPQQPYPQ), DQ8-glia-α1/ DQ8.5-glia-α1 (QGSFQPSQQ) (Sollid et al. 2012) and 33-mer toxic peptide (LQLQPFPQPQLPYPQPQLPYPQPQLPYPQPQPF) (Shan et al. 2002)

^c^Spots in which the protein was the predominant protein identified are underlined

^d^Calculations can be found in Supplementary file 3

^e^Transcriptome data summarized from Huo et al. 2018b and expressed as fragments per kilobase per million mapped reads (FPKM)

^f^Also contains 33-mer toxic peptide

Two full-length delta gliadin genes in Chinese Spring encode proteins with predicted sizes of 34.6 to 35.5 kDa and pIs from 6.37 to 7.13 (Table 2). These would be expected to overlap with some of the alpha gliadins on 2-D gels. Most gamma gliadins in Chinese Spring also overlap with alpha gliadins in size, but generally are more basic with pIs from 7.08 to 8.51. Gamma-A1 and -B4 with predicted sizes of 36.9 and 39.9 kDa, respectively, are larger than other gamma gliadins and most alpha gliadins, while gamma-A3/A4, encoded by two adjacent genes, and gamma-D4 are the most basic gamma gliadins. Three gamma gliadins also contain poly Q regions (gamma-A1, -B4, -D2) that range from 10 to 15 residues. Both delta gliadins and eight of the 11 gamma gliadins were also found in the dataset of Juhász et al. (2018) (Table 2).

**Table 2:** Characteristics of proteins deduced from full-length delta, gamma, and omega gliadin and LMW-GS genes reported by Huo et al. (2018a) in Chinese Spring, comparison with proteins deduced from sequences reported by Juhász et al. (2018), protein spot numbers and accumulation levels in 2-D gels, and comparison with transcript levels reported by Huo et al. (2018a)

Gene	N-terminal sequence	Predicted MW^a^	Predicted pI^a^	# cys	Longest poly Q^b^	# CD epitopes^c^	# WDEIA epitopes^d^	Included in Juhász et al. (2018)	2-D spot numbers^e^	% total normalized spot volume^f^	FPKM at 20 DPA^g^
δ-B1	IVQL	34,558	7.13	8		0	0	x			283
δ-D1	QLDP	35,450	6.37	8		0	0	x	80, 81, 82	0.9	3303
γ-A1	NIQ	36,892	7.73	8	15	10	0		61, 77, 78	1.9	15,774
γ-A3^h^	NMQ	30,655	8.50	8		5	0	x	123, 124, 125, 126 ^h^	2.9^f^	9073
γ-A4^h^	NMQ	30,655	8.50	8		5	0	x	123, 124, 125, 126 ^h^	2.9^f^	12,897
γ-B1	NMQ	32,431	7.16	9		8	0	x	99	0.4	21,494
γ-B2	NMQ	31,834	8.21	8		6	0	x	102	0.4	8704
γ-B4	NMQ	39,892	8.22	8	14	10	0		74, 75, 76, 78	1.9	36,657
γ-B6	NMQ	30,982	8.20	8		5	0	x	121, 122, 123, 139	0.8	21,752
γ-D1	NMQ	32,606	8.17	9		7	0		99, 100, 102, 107	1.1	14,749
γ-D2	NIQ	35,188	8.22	8	10	10	0	x	86, 101	1.7	34,746
γ-D3	NMQ	31,619	7.08	8		6	0	x	109	0.3	11,251
γ-D4	NMQ	31,865	8.51	8		6	0	x			828
ω-A4^i^	ARQ	39,651	9.69	0		3	0	x	37, 75, 79	1.1	7425
ω-B3	SRL	47,650	6.16	0		0	26				3547
ω-B6	SRL	51,532	6.01	0		0	29		25, 27, 28, 29	6.3	13,288
ω-D1	ARQ	41,831	5.02	1		12	0		34, 35	0.5	10,341
	KELQ^j^	40,950	4.68	1		12	0				
ω-D2^h,j^	ARE	42,744	5.45	0		18	0	x	31, 32^h^	3.5^f^	14,548
	KELQ^j^	41,862	5.40	0		18	0				
ω-D3^h,j^	ARE	42,744	5.45	0		18	0	x	31, 32^h^	3.5^f^	14,532
	KELQ^j^	41,862	5.40	0		18	0				
ω-D4^i^	TRQ	44,416	6.75	0		0	33		43	0.3	916
LMW-A2	ISQQQ	41,267	8.49	8	12^j^	1	0		63, 64, 65	2.2	23,950
LMW-B2	SHIP^k^	39,630	8.72	8		1	0		64, 66, 67, 68, 74	3.7	37,625
LMW-B3	SHIP^k^	38,573	8.92	8	23	2	1	x	69, 70	1.9	20,860
LMW-B4	METSHIP	37,807	8.47	8		0	1	x	88, 89, 90, 92	1.1	5842
LMW-D1	SHIP^k^	37,650	8.48	8		2	0	x	71, 72, 73, 75, 76	2.2	21,719
LMW-D2	METRCIP	32,879	8.72	8		1	0	x	96, 97, 98, 99	3.4	19,179
LMW-D3	METSCIP	31,773	8.88	8		0	0	x	94, 95	0.9	2613
LMW-D6	METSRV	37,735	8.17	8	10	3	1	x	87, 88, 89	1.4	13,688
LMW-D7	METSCIS	32,047	7.56	8		1	0	x			2228
LMW-D8	METSHIP	39,634	8.46	8		0	0	x	72, 73	0.5	6539

^a^Mature protein following removal of signal peptide

^b^Only polyQ tracks longer than 6 amino acids are reported

^c^CD-relevant epitopes summarized by Sollid et al. (2012) include DQ2.5-glia-γ1/DQ8-glia-γ1 (PQQSFPQQQ), DQ2.5-glia-γ2 (IQPQQPAQL), DQ2.5-glia-γ3/DQ8-glia-γ1b (QQPQQPYPQ), DQ2.5-glia-γ4a (SQPQQQFPQ), DQ2.5-glia-γ4b (PQPQQQFPQ), DQ2.5-glia-γ4c/DQ8-glia-γ1a (QQPQQPFPQ), DQ2.5-glia-γ4d (PQPQQPFCQ), DQ2.5-glia-γ5 (QQPFPQQPQ), DQ2.5-glia-ω1 (PFPQPQQPF), DQ2.5-glia-ω2 (PQPQQPFPW), DQ2.5-glut-L1 (PFSQQQQPV), and DQ2.5-glut-L2 (FSQQQQSPF). Glutamine residues deamidated by transglutaminase 2 may differ in DQ2.5 and DQ8 epitopes

^d^Dominant epitopes involved in WDEIA, the serious food allergy wheat-dependent exercise-induced anaphylaxis were identified by Matsuo et al. (2004) and include QQIPQQQ, QQFPQQQ, QQSPEQQ and QQSPQQQ

^e^Spots in which the protein was the predominant protein identified are underlined

^f^Calculations can be found in Supplementary file 3

^g^Data summarized from Huo et al. 2018a

^h^Genes encode identical proteins; the same spot numbers and % total normalized spot volumes are reported for both genes

^i^Stop codon near 3′ end of gene results in truncated protein. Considered pseudogene in Huo et al. 2018a

^j^Second N-terminal sequence observed as a result of processing with an asparingyl protease

^k^Also contains a 11 amino acid polyQ region

Omega gliadins are larger than other gliadins, from 41.0 to 51.5 kDa. Most are also more acidic than the other gliadins with pIs from 4.68 to 6.16. N-terminal sequencing suggests that some omega-1,2 gliadins may undergo processing with an asparingyl protease in addition to signal peptide cleavage (Kasarda et al. 1983, Dupont et al. 2004). As a result, two proteins with slightly different sizes and pIs are the predicted gene products from omega-D1, -D2, and -D3. The sequences of two other omega gliadins, omega-A4, and -D4 are included in Table 2 although the corresponding gene sequences contain stop codons close to the 3′ end that would result in truncated proteins and thus were classified as pseudogenes in Huo et al. (2018b). However, omega-A4 has expression levels that are similar to some full-length genes and there is recent evidence from proteomic studies that a protein similar to omega-D4 is expressed in several Korean wheat cultivars (Altenbach et al. 2018). Proteins encoded by omega-A4 and -D4 are more basic than other omega gliadins. Juhász et al. (2018) reported sequences identical to three of the omega gliadins, omega-A4, -D2, and -D3.

It is notable that several gliadins contain an odd number of cysteine residues that may enable these proteins to be incorporated into the glutenin polymer. Two alpha gliadins, encoded by alpha-D6 and -D8, contain seven cysteines instead of the usual six, two gamma gliadins, gamma-B1, and -D1, contain nine cysteines instead of the usual eight, and omega-D1 contains a single cysteine.

Based on their deduced sequences, the LMW-GS would be expected to overlap with some gamma gliadins as well as a few alpha gliadins in 2-DE. The ten proteins in Chinese Spring range in size from 31.8 to 41.3 kDa and have pIs from 7.56 to 8.88. Three of these proteins, LMW-B2, -B3, and -D1, likely undergo N-terminal processing by an asparaginyl endoprotease in addition to signal peptide removal and thus are expected to begin with SHIP rather than MENSHIP (d’Ovidio and Masci 2004). Three LMW-GS contain poly Q regions; LMW-A2 contains two regions of 11 and 12 amino acids, while LMW-B3 and -D6 contain poly Q regions of 23 and 10 residues, respectively. Eight of the ten LMW-GS were reported in the dataset of Juhász et al. (2018).

### 2-DE and MS/MS analysis of flour proteins

**Fig. 1:**
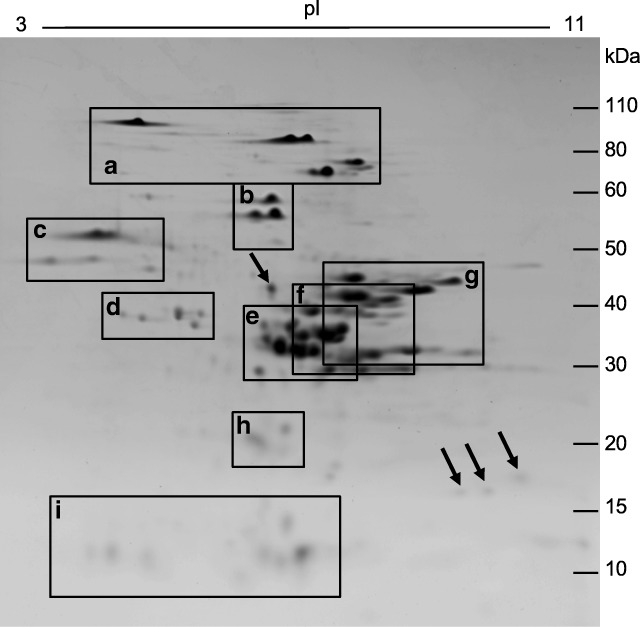
2-DE separation of total flour proteins from Chinese Spring. The positions of major protein groups are indicated by boxes: a) HMW-GS; b) omega-5 gliadins; c) omega-1,2 gliadins; d) serpins; e) alpha gliadins; f) gamma gliadins; g) LMW-GS; h) purinins; and i) alpha-amylase/protease inhibitors (AAI). Arrows point to protein spots identified as triticins

A total protein extract from Chinese Spring flour was separated by 2-DE and 166 spots were excised from gels for MS/MS analysis (Supplementary Fig. 2). The initial search of MS data was against a database containing non-redundant Triticeae sequences from NCBI plus full-length gluten protein sequences from Chinese Spring (Huo et al. 2018a, b), Butte 86 (Dupont et al. 2011) and Xioayan 81(Wang et al. 2017). In this analysis, 71 spots were identified as non-gluten proteins, 16 spots were identified as HMW-GS, and 71 spots contained either gliadins or LMW-GS. Eight minor spots did not yield valid identifications (Supplementary File 1). The positions of the major protein groups in 2-DE are indicated in Fig. 1. Fifty-four different gliadin and LMW-GS sequences were identified in the analysis (Supplementary File 1). Twenty-four were from Chinese Spring, 27 from NCBI, two from Xiaoyan 81, and one from Butte 86. Nine of the NCBI sequences were partial protein sequences missing either signal peptides or small regions at the C-termini. It is likely that these sequences were preferentially selected because they had higher sequence coverage than the homologous sequences from Chinese Spring. Twenty-six of the 27 NCBI sequences, one Xiaoyan 81 sequence, and the Butte 86 sequence were good matches to Chinese Spring sequences. In the overall analysis, 35 of the Chinese Spring gene sequences, including 15 alpha, one delta, seven gamma and three omega gliadins, and nine LMW-GS could be associated with specific protein spots.

**Fig. 2:**
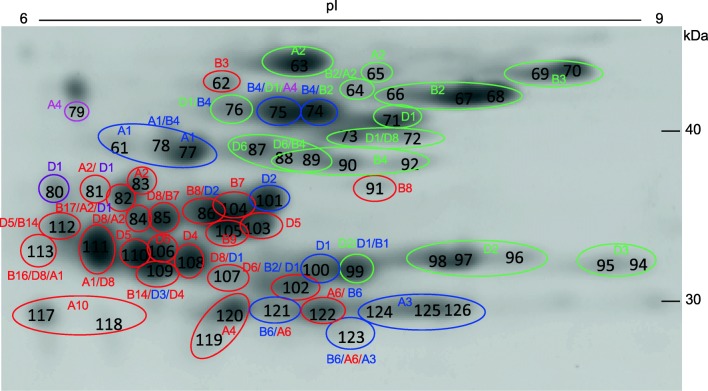
Region of the 2-D gel containing most alpha, gamma, and delta gliadins and LMW-GS (boxes e, f, g in Fig. 1). 2-DE spots in which the predominant protein was identified as an alpha gliadin are shown in red, gamma gliadin in blue, delta gliadin in purple, omega gliadin in magenta, and LMW-GS in green with MS/MS identifications shown in the same colors. Multiple adjacent spots with the same identification are shown in ovals. Spot numbers are indicated in black and correspond to those in Supplementary Fig. 1. Spots 114 and 139 are minor spots and fall outside the boundaries of the figure

**Fig. 3:**
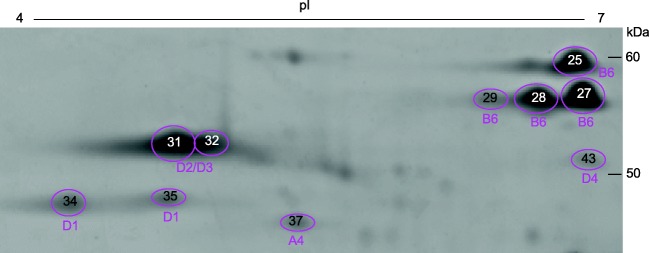
Region of the 2-D gel containing omega gliadins (boxes b, c in Fig. 1). 2-DE spots and MS/MS identifications are shown in magenta. Spot numbers are indicated inside circles and correspond to those in Supplementary Fig. 1. Spot 32 contained protein disulfide isomerase and beta-amylase in addition to an omega gliadin (Supplementary Table 1)

A second search of MS data from the spots identified as gliadins or LMW-GS was then performed against a database containing only the gluten protein sequences from Chinese Spring and common MS contaminant sequences (Table 3, Supplementary File 2). Figure 2 shows the region of the 2-D gel containing spots in which the predominant proteins were alpha gliadins (red), gamma gliadins (blue), delta gliadins (purple), omega gliadins (magenta), or LMW-GS (green) while Fig. 3 highlights the region of the gel containing most spots identified as omega gliadins. Alpha gliadins were the predominant proteins in 26 spots, gamma gliadins in 13 spots, omega gliadins in 11 spots, delta gliadins in 1 spot, and LMW-GS in 23 spots. Forty gluten protein gene sequences from Chinese Spring were associated with protein spots, including 16 of 26 alpha gliadins, 10 of 11 gamma gliadins, six of seven omega gliadins, one of two delta gliadins, and nine of ten LMW-GS. Minor proteins corresponding to both omega gliadin genes encoding truncated proteins (omega-A4 and -D4) were identified. Proteins were identified that corresponded to 16 of the genes that were present in the Huo et al. (2018a, b) dataset but not the Juhász et al. (2018) dataset (Tables 1 and 2).

**Table 3:** Predominant gliadins and LMW-GS in 2-DE spots from Chinese Spring determined by MS/MS

Spot number	Predominant protein	# Unique peptides	# Spectra	% Coverage	Other protein^a^	# Unique peptides	# Spectra	% Coverage	Other protein^a^	# Unique peptides	# Spectra	% Coverage
25	CS-omega-B6	113	220	84								
27	CS-omega-B6	91	192	77								
28	CS-omega-B6	55	146	54								
29	CS-omega-B6	13	28	29								
31	CS-omega-D2/D3	27	62	49								
32^b^	CS-omega-D2/D3	19	43	24								
34	CS-omega-D1	36	100	64								
35	CS-omega-D1	29	74	55								
37	CS-omega-A4	4	11	14								
43^c^	CS-omega-D4	13	33	21								
61^b^	CS-gamma-A1	10	22	19								
62	CS-alpha-B3	28	49	46								
63	CS-LMW-A2	151	441	82								
64	CS-LMW-B2	45	120	68	CS-LMW-A2	35	82	70				
65	CS-LMW-A2	55	99	68								
66	CS-LMW-B2	87	246	78								
67	CS-LMW-B2	106	322	81								
68	CS-LMW-B2	98	219	82								
69	CS-LMW-B3	61	223	67								
70	CS-LMW-B3	71	158	77								
71	CS-LMW-D1	94	206	81								
72	CS-LMW-D1	42	91	67	CS-LMW-D8	32	59	54				
73	CS-LMW-D1	59	151	76	CS-LMW-D8	40	94	67				
74	CS-gamma-B4	71	170	56	CS-LMW-B2	44	104	69				
75	CS-gamma-B4	82	169	58	CS-LMW-D1	46	97	70	CS-omega-A4	42	94	77
76	CS-LMW-D1	33	74	60	CS-gamma-B4	29	67	46				
77	CS-gamma-A1	55	121	53								
78^b^	CS-gamma-A1	20	55	33	CS-gamma-B4	17	42	41				
79^b^	CS-omega-A4	6	7	18								
80	CS-delta-D1	37	114	40								
81	CS-alpha-A2	27	48	47	CS-delta-D1	26	60	42				
82	CS-alpha-B17	32	96	51	CS-alpha-A2	22	54	53	CS-delta-D1	17	48	39
83	CS-alpha-A2	11	17	30								
84	CS-alpha-D8	45	101	62	CS-alpha-A2	31	61	66				
85	CS-alpha-D8	65	140	72	CS-alpha-B7	33	93	76				
86	CS-alpha-B8	60	135	62	CS-gamma-D2	38	76	59				
87	CS-LMW-D6	59	113	66								
88	CS-LMW-D6	42	117	61	CS-LMW-B4	21	49	51				
89	CS-LMW-D6	36	76	59	CS-LMW-B4	28	51	52				
90	CS-LMW-B4	72	138	73								
91	CS-alpha-B8	45	127	70								
92	CS-LMW-B4	31	63	43								
94	CS-LMW-D3	25	59	49								
95	CS-LMW-D3	56	113	69								
96	CS-LMW-D2	56	113	67								
97	CS-LMW-D2	79	219	74								
98	CS-LMW-D2	89	276	74								
99	CS-LMW-D2	53	139	65	CS-gamma-D1	48	146	51	CS-gamma-B1	28	66	58
100	CS-gamma-D1	24	50	44								
101	CS-gamma-D2	50	117	65								
102	CS-alpha-D6	42	109	69	CS-gamma-B2	37	85	44	CS-gamma-D1	21	72	48
103	CS-alpha-D5	96	189	83								
104	CS-alpha-B7	110	240	85								
105	CS-alpha-B9	94	199	78								
106	CS-alpha-D5	60	120	63								
107	CS-alpha-D8	24	45	51	CS-gamma-D1	18	39	26				
108	CS-alpha-D4	96	238	77								
109	CS-alpha-B14	40	91	49	CS-gamma-D3	26	52	49	CS-alpha-D4	26	51	48
110	CS-alpha-D5	82	157	71								
111	CS-alpha-A1	86	183	81	CS-alpha-D8	69	135	68				
112	CS-alpha-D5	64	126	61	CS-alpha-B14	34	82	70				
113	CS-alpha-B16	44	89	62	CS-alpha-D8	28	44	64	CS-alpha-A1	25	48	56
114^b^	CS-alpha-D8	8	12	25	CS-alpha-A1	5	7	27				
117	CS-alpha-A10	64	129	72								
118	CS-alpha-A10	17	27	49								
119^b^	CS-alpha-A4	32	78	55								
120	CS-alpha-A4	90	186	77								
121	CS-gamma-B6	54	98	67	CS-alpha-A6	34	55	68				
122	CS-alpha-A6	63	112	79	CS-gamma-B6	56	111	69				
123	CS-gamma-B6	14	32	48	CS-alpha-A6	12	24	40	CS-gamma-A3	12	29	36
124	CS-gamma-A2/A3	26	45	45								
125	CS-gamma-A2/A3	55	116	67								
126	CS-gamma-A2/A3	43	115	56								
139^b^	CS-gamma-B6	2	4	11								

^a^Other proteins identified in the same spot are reported only if the number of unique peptides assigned to a protein is greater than or equal to half the number of unique peptides assigned to the predominant protein

^b^Predominant protein in spot was a non-gluten protein (Supplementary Table 1)

^c^Spot did not yield an identification in the MS/MS experiment reported in Supplementary Table 1. Identification obtained in second MS/MS experiment where Scaffold thresholds were 99% protein, 5 peptides, 95% 20 ppm peptide

### Protein accumulation levels in flour

Gluten proteins comprised 68.5% of the total normalized spot volume of the Chinese Spring flour proteins and were equally split between gliadins and glutenins. Alpha, gamma, and omega gliadins comprised 12.0%, 9.9%, and 11.2% of the total while delta gliadins accounted for only 1.2%. LMW-GS were more abundant than HMW-GS, comprising 17.3% of the spot volume as opposed to 12.4% for the HMW-GS (Table 4, Supplementary File 3). Among the HMW-GS, the 1Dx, and 1Dy subunits encompassed 3.0 and 3.1%, while the 1Bx and 1By subunits accounted for 4.5 and 1.8% of the total spot volume, respectively (Supplementary File 3). Chain-terminating gliadins accounted for 4.5% of the total spot volume and were included in the glutenin fraction because of their ability to link into the polymer. Major groups of non-gluten proteins were the alpha-amylase and protease inhibitors (AAI) comprising 10.9%, globulins and triticins at 4.7% and 2.6%, respectively, serpins at 2.5%, and purinins at 1.5% of total protein.

**Table 4:** Protein composition of Chinese Spring flour

Protein type	% total normalized spot volume
Alpha gliadins	12.0
Gamma gliadins	9.9
Omega gliadins	11.2
Delta gliadins	1.2
Chain-terminating gliadins^a^	4.5
LMW-GS	17.3
HMW-GS	12.4
Purinins	1.5
Globulins	4.7
Triticins	2.6
Serpins	2.5
AAI	10.9
Other non-gluten/not identified	9.4

^a^Gliadins that contain odd numbers of cysteines; includes alpha-D6 and -D8, gamma-B1 and -D1, and omega-D1

Accumulation levels of individual gluten proteins identified in Chinese Spring flour are shown in Tables 1 and 2. Within each protein group, there was a range of accumulation levels. The most abundant protein in the flour was the omega-5 gliadin encoded by omega-B6, accounting for 6.3% of total flour protein. Two LMW-GS, LMW-B2, and -D2 were also abundant proteins, each accounting for more than 3% of the total protein. An omega-1,2 gliadin and a gamma gliadin also accounted for 3.5 and 2.9% of the total protein, respectively. However, both proteins are encoded by multiple genes (Table 2). The omega-1,2 gliadin is encoded by adjacent but identical genes omega-D3 and -D4 while the gamma gliadin is encoded by adjacent genes gamma-A3 and -A4 that differ by a single base pair. At the other end of the spectrum, 11 alpha gliadins, four gamma gliadins, two omega gliadins, and two LMW-GS were present at low levels in the flour, encompassing less than 1% of the total protein. Three gliadins containing extra cysteine residues (alpha-D6, gamma-B1, and omega-D1) were accumulated at low levels in Chinese Spring flour (0.4–0.5% of gluten protein) while two others, gamma-D1, and alpha-D8, encompassed 1.1 and 2.1% of the total gluten protein. With the exception of omega-D1, proteins that contained the greatest numbers of epitopes involved in celiac disease (alpha-D5, gamma-A1, -B4, -D2, and omega-D2/D3) were accumulated at moderate levels (1.7–3.5% of gluten protein) while proteins that contained epitopes for the serious food allergy wheat-dependent exercise-induced anaphylaxis WDEIA (omega-B6) were accumulated to high levels (6.3% of gluten protein) (Tables 1 and 2).

### Comparison of transcript levels and protein accumulation

Huo et al. (2018a, b) reported a wide range of transcript levels among individual members of the different gluten protein classes. The genes expressed at the highest levels in 20 DPA endosperm were gamma-B4 and -D2 and LMW-B2, all with FPKM values greater than 34,000 (Tables 1 and 2). Of the alpha gliadins, alpha-B9 and -D5 were expressed at the highest levels with FPKM values around 24,000 and 27,000, respectively. Two gamma gliadins, gamma-A3/A4 and -B1, and three other LMW-GS, LMW-A2, -B3, and -D1, had FPKM values greater than 20,000. In comparison, eight alpha gliadin genes had levels of transcripts that were less than 4000 FPKM. These included alpha-A5, -A8, -B17, -B18, -B25, -D1, -D8, and -D9. One gamma gliadin, gamma-D4, two omega gliadins, omega-B3 and -D4, two delta gliadins, and two LMW-GS, LMW-D3 and -D7, were also expressed at low levels. Only five of the genes expressed at low levels were linked to spots in 2-D gels, alpha-B17 and -D8, delta-D1, omega-D4, and LMW-D3. Additionally, there were ten genes for which proteins were not identified in the analysis, including seven alpha gliadins, one gamma gliadin, one omega gliadin and one LMW-GS. These genes accounted for only 10% of the total gliadin and LMW-GS transcripts. Seven of these genes had FPKM values less than 4000.

For many of the individual gluten protein genes, transcript levels reflected protein levels. Overall, the best accordance was for the LMW-GS. The biggest discrepancy between transcript and protein levels was for omega-B6. This omega gliadin was accumulated to the highest levels in Chinese Spring flour (6.3% of total gluten protein, but, surprisingly, FPKM levels were only moderate, around 13,000 (Table 2). Alpha-D8 had very low FPKM levels (~ 2000) but was the most abundant alpha gliadin accumulated in the flour at 2.1% of the total gluten protein. Conversely, alpha-B7 and -B9 had FPKM values from 19,000 to 24,000, but each of the proteins comprised less than 1% of the flour protein. One gamma gliadin gene, gamma-B1, also had FPKM values over 20,000 but comprised less than 1% of the total flour protein.

**Fig. 4:**
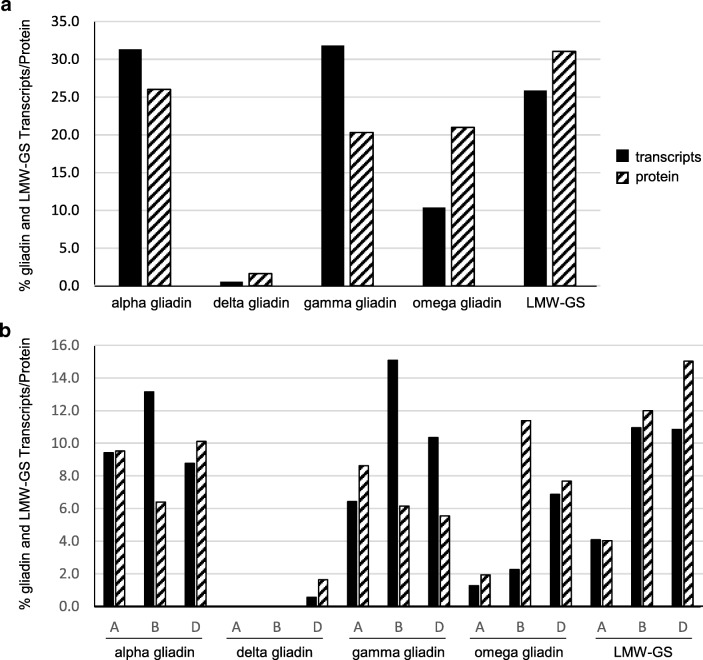
Comparison of transcript and protein levels for gliadins and LMW-GS in Chinese Spring. Panel a shows the different classes of genes and proteins while panel b shows transcript and protein levels for genes encoded by the A, B, and D genomes. Only transcript levels for gliadin and LMW-GS genes linked to proteins in 2-DE were considered in the analysis. Black bars denote transcript levels while hatched bars denote protein levels

Of the gliadin and LMW-GS genes that were linked to proteins in 2-DE in this study, alpha and gamma gliadins and LMW-GS encompassed 31, 32, and 26% of the transcripts, respectively. Omega gliadins encompassed only 10% of the transcripts. In comparison, LMW-GS accounted for 31% of the identified gliadin and LMW-GS proteins, followed by alpha gliadins at 26%, omega gliadins at 21%, and gamma gliadins at 20% (Fig. 4A). Differential accumulation of transcripts and proteins was also observed among the A, B, and D genomes (Fig. 4B). Among the alpha gliadins, the B genome contributed the highest percentage of transcripts (13.2%) but the least amount of protein (6.4%). Similarly, the B genome contributed the greatest amount of gamma gliadin transcripts (15.1%), but a greater percentage of the gamma gliadin protein was contributed by the A genome (8.6%). In comparison, the D genome was responsible for a greater percentage of the omega gliadin transcripts (6.9%), but the B genome contributed the most to protein levels (11.4%). For the LMW-GS, both transcript (10.9, 10.8%) and protein levels (12, 15%) of the B and D genomes were higher than those of the A genome (4.1%, 4%) which is not surprising since only one full-length LMW-GS gene was identified from the A genome.

## Discussion

In this paper, individual gluten proteins in flour from the reference wheat Chinese Spring were linked to a complete set of gluten protein genes annotated from the same cultivar. Total flour proteins from Chinese Spring were separated by 2-DE, digested separately with chymotrypsin, thermolysin, or trypsin and analyzed by MS/MS. Chymotrypsin and thermolysin were used to obtain maximum sequence coverage of gluten proteins while trypsin was used to optimize identification of non-gluten proteins in the flour. While the migrations of gluten proteins in the 2-D gels were generally consistent with the calculated MW and pI values, the actual proteomic map of Chinese Spring flour was considerably more complicated than what might have been expected based simply on the predicted characteristics of the deduced proteins. This was due to a number of factors. First, many adjacent spots in the 2-D gel were identified as the same proteins. Before the complete genomic sequence of Chinese Spring became available, it was thought that the numbers of expressed gluten protein genes in individual cultivars were much higher. As a result, it seemed logical that some of these spots might be the products of closely related genes. This is clearly not the case. Rather, the multiple spots are likely due to charge trains that often are observed in 2-DE. It has often been proposed that charge trains represent artifacts of the separation technique. However, there is no evidence that deamidation or carbamylation, protein modifications that would result in pI shifts, occur under normal sample handling procedures (Righetti 2006). It also has been suggested that charge trains might be prevented by alkylation of proteins prior to 2-DE. However, reduction and alkylation of wheat flour proteins did not result in fewer 2-DE spots in the present study (data not shown) or in the study of Dupont et al. (2011) using the cultivar Butte 86. As discussed by Dupont et al. (2011), the multiple spots may be due to protein modifications caused by naturally occurring in vivo processes. However, the biological significance of such modifications, if any, remains to be determined.

It is also apparent from the analysis that some gluten proteins do not migrate on 2-D gels according to their molecular weights. For example, spots 69 and 70 were identified as LMW-B3 with a predicted MW of 38.6 kDa and a pI of 8.92. Interestingly, spots 66, 67, and 68, identified as LMW-B2, run ahead of spots 69 and 70 in the second dimension of the 2-D gel even though the predicted MW of LMW-B2 is greater than that of -B3 (Fig. 2, Table 2). One possibility is that LMW-B2 undergoes post-translational processing. Thus far, N-terminal processing by an asparaginyl endopetidase has been described for only a few omega gliadins and the s-type LMW-GS (Dupont et al. 2004) while evidence for C-terminal processing has been presented for certain HMW-GS (Nunes-Miranda et al. 2017). Both LMW-B2 and -B3 are s-type LMW-GS and subject to similar proteolytic processing at their N-termini. Additionally, peptides corresponding to the C-termini were obtained by MS/MS for both LMW-B2 and -B3 (Supplementary File 2), suggesting that it is unlikely that differential C-terminal processing accounts for the discrepancy in size. An alternate explanation lies in the structure of the two proteins. While the sequences are quite similar overall, LMW-B3 includes a 23 amino acid poly Q region not found in LMW-B2 (Supplementary Fig. 3). It is possible that this poly Q region affects the mobility of the protein by influencing either the shape of the protein or its binding to SDS.

Not surprisingly, many spots on the 2-D gels contained more than one protein. This would be expected since many gliadins and LMW-GS have very similar MWs and pIs. In our analysis, 18 spots contained two proteins while seven spots contained three proteins. For example, spot 109 contained two alpha gliadins, alpha-B14 and -D4, and one gamma gliadin, gamma-D3. These proteins range from 31.4 to 31.6 kDa and have predicted pIs from 6.79 to 7.08. We were unable to identify proteins corresponding to all of the full-length genes. Some of these genes were expressed at low levels in the endosperm and might be expected to be minor proteins. Others encoded proteins with high similarity to identified proteins that may not have been distinguished in the MS analysis.

The complexity of gluten protein fractions in 2-DE has been the subject of a number of other studies. Kawaura et al. (2018) resolved 70 spots by 2-DE from a gliadin fraction from Chinese Spring. Using aneuploid lines, they mapped 10 spots to chromosome 6A, 10 to 6B, and 16 to 6D, suggesting that these spots correspond to alpha gliadins. They also mapped six spots to chromosome 1A, three to 1B, and seven to 1D, suggesting that these spots correspond to gamma gliadins. In addition, 18 spots were present in all aneuploid lines and likely consisted of overlapping proteins that were encoded on more than one chromosome. The authors did not attempt to link protein spots to specific gene sequences in their study. However, by N-terminal sequencing, they determined that 48 spots corresponded to alpha gliadins and 22 spots corresponded to gamma gliadins. Taking into consideration the numbers of spots and the numbers of full-length genes revealed by genomic sequencing (Huo et al. 2018a, b), it is likely that the same protein sequence would be found in multiple 2-DE spots.

Wang et al. (2017) also associated 82 2-DE spots from a gliadin fraction from the cultivar Xioayan 81 with DNA sequences for 25 alpha, 11 gamma, 1 delta, and 5 omega gliadins that were obtained from the same cultivar through RNA sequencing experiments. More than half of the 42 genes were associated with more than one protein spot and, in some cases, as many as seven spots were identified as the same protein. In another recent study, Cho et al. (2018) examined gliadins in the Korean wheat Keumkang, although corresponding gene sequences from this cultivar were not available. Of 98 spots in a gliadin fraction, 31 were identified as alpha gliadins, 28 as gamma gliadins, and one as an omega gliadin. Again, multiple 2-DE spots, in one case as many as 11, yielded the same identification by MS/MS. As discussed by Dupont et al. (2011), it is critical to know that multiple 2-DE spots contain the same protein when interpreting the results of comparative proteomic experiments.

The previous studies (Kawaura et al. 2018; Wang et al. 2017; Cho et al. 2018) focused specifically on gliadin fractions from the flour. This reduced the numbers of proteins and made it possible to use a tighter pH range of 6–11 for the first-dimension separation. However, the use of fractionated proteins also introduces a number of potential problems. First, some gliadins partition into other fractions. Gliadins that contain an odd number of cysteine residues preferentially partition with the polymeric glutenins instead of the monomeric gliadins (Vensel et al. 2014). In fact, these gliadins were found in the greatest abundance in fractions containing small glutenin polymers, supporting the notion that they serve as chain terminators of the polymer and could impact end-use quality. Additionally, some omega-1,2 gliadins are found in abundance in salt-soluble protein fractions (Hurkman and Tanaka 2004). Depending on the extraction method, other gluten and non-gluten proteins also may be found in the gliadin fraction. In the study of Cho et al. (2018), LMW-GS comprised 12% and non-gluten proteins such as purinins and alpha amylase inhibitors comprised 6% of the total spot volume of the gliadin fraction. The current study focused on the separation and identification of total flour proteins rather than gliadin and glutenin protein fractions because environmental factors and genetic modifications affect the flour proteome in profound ways and it is important to consider the entire complement of flour proteins for meaningful biological studies.

While transcript levels for some genes were in accordance with protein levels, this was not always the case, suggesting that transcriptomic analyses of developing endosperm can complement but not substitute for proteomic analyses of the flour. Discrepancies between transcript and protein levels may be due to the growth conditions of the plant material if RNA and protein levels are not determined from material grown at the same time. Indeed, environmental conditions under which the grain is produced have a profound impact on both gene expression and the levels of certain gluten proteins (Altenbach et al. 2011a; Altenbach 2012, Juhász et al. 2018). It also is important to keep in mind that the stringency of parameters used in assembling closely related and repetitive gene sequences genes can influence the results of transcriptomic studies. This is particularly true for the omega gliadins that consist almost entirely of repetitive sequences and may explain the large discrepancy between transcript and protein levels for the omega gliadins encoded by the B genome. Observed discrepancies between transcript levels and protein levels of gluten proteins encoded by the A, B, and D genomes demonstrate that there remains much to be learned about the regulation of these genes and proteins in hexaploid wheat.

Ultimately, the complement of specific gluten proteins and the amounts of individual proteins in the flour are important for determining both quality and immunogenic potential. Because Chinese Spring has poor flour quality and is not grown commercially, there is a need to extend the findings from this reference cultivar to commercial cultivars grown in various parts of the world. While the specific cultivars will vary from country to country, the reference genome sequence from Chinese Spring now makes it possible to design gene capture methods to select genomic regions containing gluten protein genes and to obtain complete sets of gluten protein gene sequences from cultivars with different end-use quality. These studies coupled with detailed proteomic analyses should provide new insight into the molecular basis of wheat flour quality and allergenic potential and facilitate new research to improve the healthfulness and end-use properties of wheat. Knowledge about the entire complement of gluten proteins also makes it possible to identify peptides unique for individual gluten proteins that can form the basis for targeted MS methods.

## Electronic supplementary material

Supplementary Figure 1 Sequence comparison of selected alpha gliadins encoded by the B genome of Chinese Spring. (DOCX 14 kb)

Supplementary Figure 2 2-DE spots that were identified by MS/MS. Panel A shows the entire 2-D gel while panel B shows an enlarged version of the region enclosed in the box. (PPTX 2021 kb)

Supplementary Figure 3 Sequence comparison of two S-type LMW-GS showing a poly Q region that distinguishes the two proteins in the red box. (PPTX 53 kb)

Supplementary File 1 Predominant proteins in 2-DE spots from Chinese Spring flour determined by MS/MS after searching a database containing non-redundant Triticeae sequences from NCBI plus selected sequences from Chinese Spring, Butte 86 and Xioayan 81. (XLSX 24 kb)

Supplementary File 2 Peptide data for individual protein spots identified by MS/MS in Table 3. Data was extracted from Scaffold. MS/MS sequence coverage for each protein is also shown. (XLSX 7009 kb)

Supplementary File 3 Normalized spot volumes determined by SameSpots software for triplicate gels of three separate protein extractions. (XLSX 121 kb)

## Abbreviations

2-DE: Two-dimensional gel electrophoresis
AAI: Alpha-amylase/protease inhibitor
FPKM: Fragments per kilobase per million mapped reads
HMW-GS: High-molecular-weight glutenin subunit
LMW-GS: Low-molecular-weight glutenin subunit
MS: Mass spectrometry
MS/MS: Tandem mass spectrometry
WDEIA: Wheat-dependent exercise-induced anaphylaxis

## References

[CR1] Altenbach SB (2012). New insights into the effects of high temperature, drought and post-anthesis fertilizer on wheat grain development. J Cereal Sci.

[CR2] Altenbach SB, Tanaka CK, Hurkman WJ, Whitehand LC, Vensel WH, Dupont FM (2011). Differential effects of a post-anthesis fertilizer regimen on the wheat flour proteome determined by quantitative 2-DE. Proteome Sci.

[CR3] Altenbach SB, Vensel WH, Dupont FM (2011). The spectrum of low molecular weight alpha-amylase/protease inhibitor genes expressed in the US bread wheat cultivar Butte 86. BMC Res Notes.

[CR4] Altenbach SB, Chang H-C, Simon-Buss A, Jang Y-R, Denery-Papini S, Pineau F, Gu YQ, Huo N, Lim S-H, Kang C-S, Lee J-Y (2018). Towards reducing the immunogenic potential of wheat flour: omega gliadins encoded by the D genome of hexaploid wheat may also harbor epitopes for the serious food allergy WDEIA. BMC Plant Biol.

[CR5] Anderson OD, Litts JC, Greene FC (1997). The α-gliadin gene family. I. Characterization of ten new wheat α-gliadin genomic clones, evidence for limited sequence conservation of flanking DNA, and southern analysis of the gene family. Theor Appl Genet.

[CR6] Cho K, Beom H-R, Jang Y-R, Altenbach SB, Vensel WH, Simon-Buss A, Lim S-H, Kim MG, Lee J-Y (2018). Proteomic profiling and epitope analysis of the complex α-, γ- and ω-gliadin families in a commercial bread wheat. Front Plant Sci.

[CR7] Dupont FM, Vensel W, Encarnacao T, Chan R, Kasarda DD (2004). Similarities of omega gliadins from *Triticum urartu* to those encoded on chromosome 1A of hexaploid wheat and evidence for their post-translational processing. Theor Appl Genet.

[CR8] Dupont Frances M, Vensel William H, Tanaka Charlene K, Hurkman William J, Altenbach Susan B (2011). Deciphering the complexities of the wheat flour proteome using quantitative two-dimensional electrophoresis, three proteases and tandem mass spectrometry. Proteome Science.

[CR9] Forde J, Malpica HM, Halford NG, Shewry PR, Anderson OD, Greene FC, Miflin BJ (1985). The nucleotide sequence of a HMW glutenin subunit gene located on chromosome 1A of wheat. (*Triticum aestivum* L.). Nucleic Acids Res.

[CR10] Huo N, Zhang S, Zhu T, Dong L, Mohr T, Hu T, Liu Z, Dvorak J, Luo M-C, Wang D, Lee J-Y, Altenbach S, Gu YQ (2018). Gene duplication and evolution dynamics in the homeologous regions harboring multiple prolamin and resistance gene families in hexaploid wheat. Front Plant Sci.

[CR11] Huo N, Zhu T, Altenbach S, Dong L, Wang Y, Mohr T, Liu Z, Dvorak J, Luo M-C, Gu YQ (2018). Dynamic evolution of α-gliadin prolamin gene family in homeologous genomes of hexaploid wheat. Sci Reports.

[CR12] Hurkman William J., Tanaka Charlene K. (2004). Improved methods for separation of wheat endosperm proteins and analysis by two-dimensional gel electrophoresis. Journal of Cereal Science.

[CR13] International Wheat Genome Sequencing Consortium (IWGSC) (2018). Shifting the limits in wheat research and breeding through a fully annotated and anchored reference genome sequence. Science.

[CR14] Juhász A, Belova T, Florides CG, Maulis C, Fischer I, Gell G, Birinyi Z, Ong J, Keeble-Gagnère G, Maharajan A, Ma W, Gibson P, Jia J, Lang D, Mayer KFX, Spannagl M, Tye-Din JA, Appels R, Olsen OA, International Wheat Genome Sequencing Consortium (2018). Genome mapping of seed-borne allergens and immunoresponsive proteins in wheat. Sci Adv.

[CR15] Kasarda DD, Autran J-C, Lew EJ-L, Nimmo CC, Shewry PR (1983). N-terminal amino acid sequences of ω-gliadins and ω-secalins: implication for the evolution of prolamin genes. Biochim Biophys Acta.

[CR16] Kawaura K, Miura M, Kamei Y, Ikeda TM, Ogihara Y (2018). Molecular characterization of gliadins of Chinese spring wheat in relation to celiac disease elicitors. Genes Genet Syst.

[CR17] Lowry OH, Rosebrough NJ, Farr AL, Randall RJ (1951). Protein measurement with the Folin phenol reagent. J Biol Chem.

[CR18] Matsuo H, Morita E, Tatham AS, Morimoto K, Horikawa T, Osuna H, Ikezawa Z, Kaneko S, Kohno K, Dekio S (2004). Identification of the IgE-binding epitope in ω-5 gliadin, a major allergen in wheat-dependent exercise-induced anaphylaxis. J Biol Chem.

[CR19] Nunes-Miranda JD, Bancel E, Viala D, Chambon C, Capelo JL, Branlard G, Ravel C, Igrejas G (2017). Wheat glutenin: the “tail” of the 1By protein subunits. J Proteome.

[CR20] Righetti Pier Giorgio (2006). Real and imaginary artefacts in proteome analysis via two-dimensional maps. Journal of Chromatography B.

[CR21] Sabelli P, Shewry PR (1991). Characterization and organization of gene families at the *Gli-1* loci of bread and durum wheats by restriction fragment analysis. Theor Appl Genet.

[CR22] Shan L. (2002). Structural Basis for Gluten Intolerance in Celiac Sprue. Science.

[CR23] Shewry PR, Halford NG, Lafiandra D (2003). Genetics of wheat gluten proteins. Adv Genet.

[CR24] Sollid LM, Qiao S-W, Anderson RP, Gianfrani C, Koning F (2012). Nomenclature and listing of celiac disease relevant gluten T-cell epitopes restricted by HLA-DQ molecules. Immunogenet.

[CR25] Tao HP, Kasarda DD (1989). Two-dimensional gel mapping and N-terminal sequencing of LMW-glutenin subunits. J Exp Bot.

[CR26] Vensel WH, Tanaka CK, Altenbach SB (2014). Protein composition of wheat gluten polymer fractions determined by quantitative two-dimensional gel electrophoresis and tandem mass spectrometry. Proteome Sci.

[CR27] Wang DW, Li D, Wang J, Zhao Y, Wang Z, Yue G, Liu X, Qin H, Zhang K, Dong L, Wang D (2017). Genome-wide analysis of complex wheat gliadins, the dominant carriers of celiac disease epitopes. Sci Rep.

[CR28] Zimin AV, Puiu D, Hall R, Kingan S, Clavijo BJ, Salzberg SL (2017). The first near-complete assembly of the hexaploid bread wheat genome, *Triticum aestivum*. GigaSci.

