# Supplementary material for: Exploiting the reference genome sequence of hexaploid wheat: a proteomic study of flour proteins from the cultivar Chinese Spring
Source: Funct Integr Genomics. 2019 Jun 27;20(1):1–16. doi: 10.1007/s10142-019-00694-z (PMC6954139; doi:10.1007/s10142-019-00694-z)
Supplement: Supplementary file 1 — Sequence comparison of selected alpha gliadins encoded by the B genome of Chinese Spring. (DOCX 14 kb) [file 10142_2019_694_MOESM1_ESM.docx]

Supplementary Figure 1. Sequence comparison of selected alpha gliadins encoded by the B genome of Chinese Spring. Sequence variations are shown in red.

CS-alpha-B7 MKTFLILALLAIVATTATTAVRVPVPQLQPQNPSQQQPQEQVPLVQQQQFPGQQQQFPPQ

CS-alpha-B9 MKTFLILALLAIVATTATTAVRVPVPQLQPQNPSQQQPQEQVPLVQQQQFPGQQQQFPPQ

************************************************************

CS-alpha-B7 QPYPQPQPFPSQQPYLQLQPFPQPQPFPPQLPYPQPQSFPPQQPYPQQQPQYLQPQQPIS

CS-alpha-B9 QPYPQPQPFPSQQPYLQLQPFPQPQPFPPQLPYPQPQSFPPQQPYPQQQPQYLQPQQPIS

************************************************************

CS-alpha-B7 QQQAQQQQQQQQQQQQQQQILQQILQQQLIPCRDVVLQQHNIAHASSQVLQQSTYQLLQQ

CS-alpha-B9 QQQAQQQQQQQQQQQQQQQILQQILQQQLIPCRDVVLQQHNIAHASSQVLQQSTYQLLQQ

************************************************************

CS-alpha-B7 LCCQQLLQIPEQSRCQAIHNVAHAIIMHQQQQQQQEQQQQLQQQQQQQLQQQRQQPSSQV

CS-alpha-B9 LCCQQLLQIPEQSRCQAIHNVAHAIIMHQQQQQQQEQQQQLQQQQQQQLHQQRQQPSSQV

*************************************************:**********

CS-alpha-B7 SFQQPQQQYPSSQVSFQPSQLNPQAQGSVQPQQLPQFAEIRNLALQTLPAMCNVYIPPHC

CS-alpha-B9 SFQQPQQQYPSSQVSFQPSQLNPQAQGSVQPQQLPQFAEIRNLALQTLPAMCNVYIPPHC

************************************************************

CS-alpha-B7 STTIAPFGIFGTN

CS-alpha-B9 STTIAPFGIFGTN

*************

CS-alpha-B18 MKTFLILALLAIVATTTTTAVRVPVPQLQPQNPSQQQPQEQVPLVQQQQFLGQQQQQFPG

CS-alpha-B15 MKTFLILALLAIMATTTTTAVRVPVPQLQPQNPSQQQPQEQVPLVQQQQFLGQQQQQFPG

CS-alpha-B17 MKTFLILALLAIVATTTTTAVRVPVPQLQPQNPSQQQPQEQVPLVQQQQFLGQQQQQFPG

CS-alpha-B16 MKTFLILALLAIVATTTTTAVRVPVPQLQPQNPSQQQPQEQVPLVQQQQFLGQQQQQFPG

CS-alpha-B11 MKTFLILALLAIVATTTTTAVRVPVPQLQPQNPSQQQPQEQVPLVQQQQFLGQQQQQFPG

CS-alpha-B14 MKTFLILALLAIVATTTTTAVRVPVPQLQPQNPSQQQPQEQVPLVQQQQFLGQQQQQFPG

************:***********************************************

CS-alpha-B18 QQQPFPPQQPYPQPQPFLPQLPYPQPQPFPPQQSYPQPQPQYPQPQQPISQQQAQLLQQQ

CS-alpha-B15 QQQPFPPQQPYPQPQPFLPQLPYPQPQPFPPQQSYPQPQPQYPQPQQPISQQQAQL----

CS-alpha-B17 QQQPFPPQQPYPQPQPFLPQLPYPQPQPFPPQQSYPQPQPQYPQPQQPISQQQAQLLQQQ

CS-alpha-B16 QQQPFPPQQPYPQPQPFLPQLPYPQPQPFPPQQSYPQPQPQYPQPQQPISQQQAQL----

CS-alpha-B11 QQQPFPPQQPYPQPQPFLPQLPYPQPQPFPPQQSYPQPQPQYPQPQQPISQQQAQL---Q

CS-alpha-B14 QQQPFPPQQPYPQPQPFLPQLPYPQPQPFPPQQSYPQPQPQYPQPQQPISQQQAQL---Q

********************************************************

CS-alpha-B18 QQQQQQQQQQQQILQQILQQQLIPCRDVVLQQPNIAHASSQVSQQSYQLLQQLCCQQLWQ

CS-alpha-B15 -QQQQQQQQQQQILQQILQQQLIPCRDVVLQQPNIAHASSQVSQQSYQLLQQLCCQQLWQ

CS-alpha-B17 QQQQQQQQQQQQILQQILQQQLIPCRDVVLQQPNIAHASSQVSQQSYQLLQQLCCQQLWQ

CS-alpha-B16 -QQQQQQQQQQQILQQILQQQLIPCRDVVLQQPNIAHASSQVSQQSYQLLQQLCCQQLWQ

CS-alpha-B11 QQQQQQQQQQQQILQQILQQQLIPCRDVVLQQPNIAHASSKVSQQSYQLLQQLCCLQLWQ

CS-alpha-B14 QQQQQQQQQQQQILQQILQQQLIPCRDVVLQQPNIAHASSKVSQQSYQLLQQLCCQQLWQ

***************************************:************** ****

CS-alpha-B18 TPEQSRCQAIHNVIHAIILH-------HRHQQQQQQQQQQQQQPSSQVSYQQPQQQYPSG

CS-alpha-B15 TPEQSRCQAIHNVIHAIILH------QQQQQQQQQQQQQQQQQPSSQVSYQQPQQQYPSG

CS-alpha-B17 TPEQSRCQAIHNVIHAIILH-----QQQQQQQQQQQQQQQQQQPSSQVSYQQPQQQYPSG

CS-alpha-B16 TPEQSRCQAIHNVIHAIILHQQQQQQQQQQQQQQQQQQQQQQQPSSQVSYQQPQQQYPSG

CS-alpha-B11 TPEQSRCQAIHNVIHAIILH------HQQQQQQQQQQQQQQQQPSSQVSYQQPQQQYPSG

CS-alpha-B14 TPEQSRCQAIHNVIHAIILH-------QQQQQQQQQQQQQQQQPSSQVSYQQPQQQYPSG

******************** :.:******************************

CS-alpha-B18 QGFFQPSQQNPQAQGFVQPQQLPQFEEIRNLALQTLPAMCNVYIPPYCSTTIAPFGIMST

CS-alpha-B15 QGFFQPSQQNPQAQGFVQPQQLPQFQEIRNLALQTLPAMCNVYIPPYCSTTIAPFGIMST

CS-alpha-B17 QGFFQPSQQNPQAQGFVQPQQLPQFEEIRNLALQTLPAMCNVYIPPYCSTTIAPFGIMST

CS-alpha-B16 QGFFQPSQQNPQAQGFVQPQQLPQFEEIRNLALQTLPAMCNVYIPPYCSTTIAPFGIMST

CS-alpha-B11 QGFFQPSQQNPQAQGFVQPQQLPQFEEIRNLALQTLPAMCNVYIPPYCSTTIAPFGIMST

CS-alpha-B14 QGFFQPSQQNPQAQGFVQPQQLPQFEEIRNLALQTLPAMCNVYIPPYCSTTIAPFGIMST

*************************:**********************************

CS-alpha-B18 N

CS-alpha-B15 N

CS-alpha-B17 N

CS-alpha-B16 N

CS-alpha-B11 N

CS-alpha-B14 N

*

CS-alpha-B11 MKTFLILALLAIVATTTTTAVRVPVPQLQPQNPSQQQPQEQVPLVQQQQFLGQQQQQFPG

CS-alpha-B14 MKTFLILALLAIVATTTTTAVRVPVPQLQPQNPSQQQPQEQVPLVQQQQFLGQQQQQFPG

************************************************************

CS-alpha-B11 QQQPFPPQQPYPQPQPFLPQLPYPQPQPFPPQQSYPQPQPQYPQPQQPISQQQAQLQQQQ

CS-alpha-B14 QQQPFPPQQPYPQPQPFLPQLPYPQPQPFPPQQSYPQPQPQYPQPQQPISQQQAQLQQQQ

************************************************************

CS-alpha-B11 QQQQQQQQQILQQILQQQLIPCRDVVLQQPNIAHASSKVSQQSYQLLQQLCCLQLWQTPE

CS-alpha-B14 QQQQQQQQQILQQILQQQLIPCRDVVLQQPNIAHASSKVSQQSYQLLQQLCCQQLWQTPE

**************************************************** *******

CS-alpha-B11 QSRCQAIHNVIHAIILHHQQQQQQQQQQQQQQQQPSSQVSYQQPQQQYPSGQGFFQPSQQ

CS-alpha-B14 QSRCQAIHNVIHAIIL-HQQQQQQQQQQQQQQQQPSSQVSYQQPQQQYPSGQGFFQPSQQ

**************** *******************************************

CS-alpha-B11 NPQAQGFVQPQQLPQFEEIRNLALQTLPAMCNVYIPPYCSTTIAPFGIMSTN

CS-alpha-B14 NPQAQGFVQPQQLPQFEEIRNLALQTLPAMCNVYIPPYCSTTIAPFGIMSTN

****************************************************

CS-alpha-B15 MKTFLILALLAIMATTTTTAVRVPVPQLQPQNPSQQQPQEQVPLVQQQQFLGQQQQQFPG

CS-alpha-B16 MKTFLILALLAIVATTTTTAVRVPVPQLQPQNPSQQQPQEQVPLVQQQQFLGQQQQQFPG

************:***********************************************

CS-alpha-B15 QQQPFPPQQPYPQPQPFLPQLPYPQPQPFPPQQSYPQPQPQYPQPQQPISQQQAQLQQQQ

CS-alpha-B16 QQQPFPPQQPYPQPQPFLPQLPYPQPQPFPPQQSYPQPQPQYPQPQQPISQQQAQLQQQQ

************************************************************

CS-alpha-B15 QQQQQQQILQQILQQQLIPCRDVVLQQPNIAHASSQVSQQSYQLLQQLCCQQLWQTPEQS

CS-alpha-B16 QQQQQQQILQQILQQQLIPCRDVVLQQPNIAHASSQVSQQSYQLLQQLCCQQLWQTPEQS

************************************************************

CS-alpha-B15 RCQAIHNVIHAIILH------QQQQQQQQQQQQQQQQQPSSQVSYQQPQQQYPSGQGFFQ

CS-alpha-B16 RCQAIHNVIHAIILHQQQQQQQQQQQQQQQQQQQQQQQPSSQVSYQQPQQQYPSGQGFFQ

*************** ***************************************

CS-alpha-B15 PSQQNPQAQGFVQPQQLPQFQEIRNLALQTLPAMCNVYIPPYCSTTIAPFGIMSTN

CS-alpha-B16 PSQQNPQAQGFVQPQQLPQFEEIRNLALQTLPAMCNVYIPPYCSTTIAPFGIMSTN

********************:***********************************
