# Supplementary material for: Exploiting the reference genome sequence of hexaploid wheat: a proteomic study of flour proteins from the cultivar Chinese Spring
Source: Funct Integr Genomics. 2019 Jun 27;20(1):1–16. doi: 10.1007/s10142-019-00694-z (PMC6954139; doi:10.1007/s10142-019-00694-z)
Supplement: Supplementary file 2 — 2-DE spots that were identified by MS/MS. Panel A shows the entire 2-D gel while panel B shows an enlarged version of the region enclosed in the box. (PPTX 2021 kb) [file 10142_2019_694_MOESM2_ESM.pptx]

## Slide 1
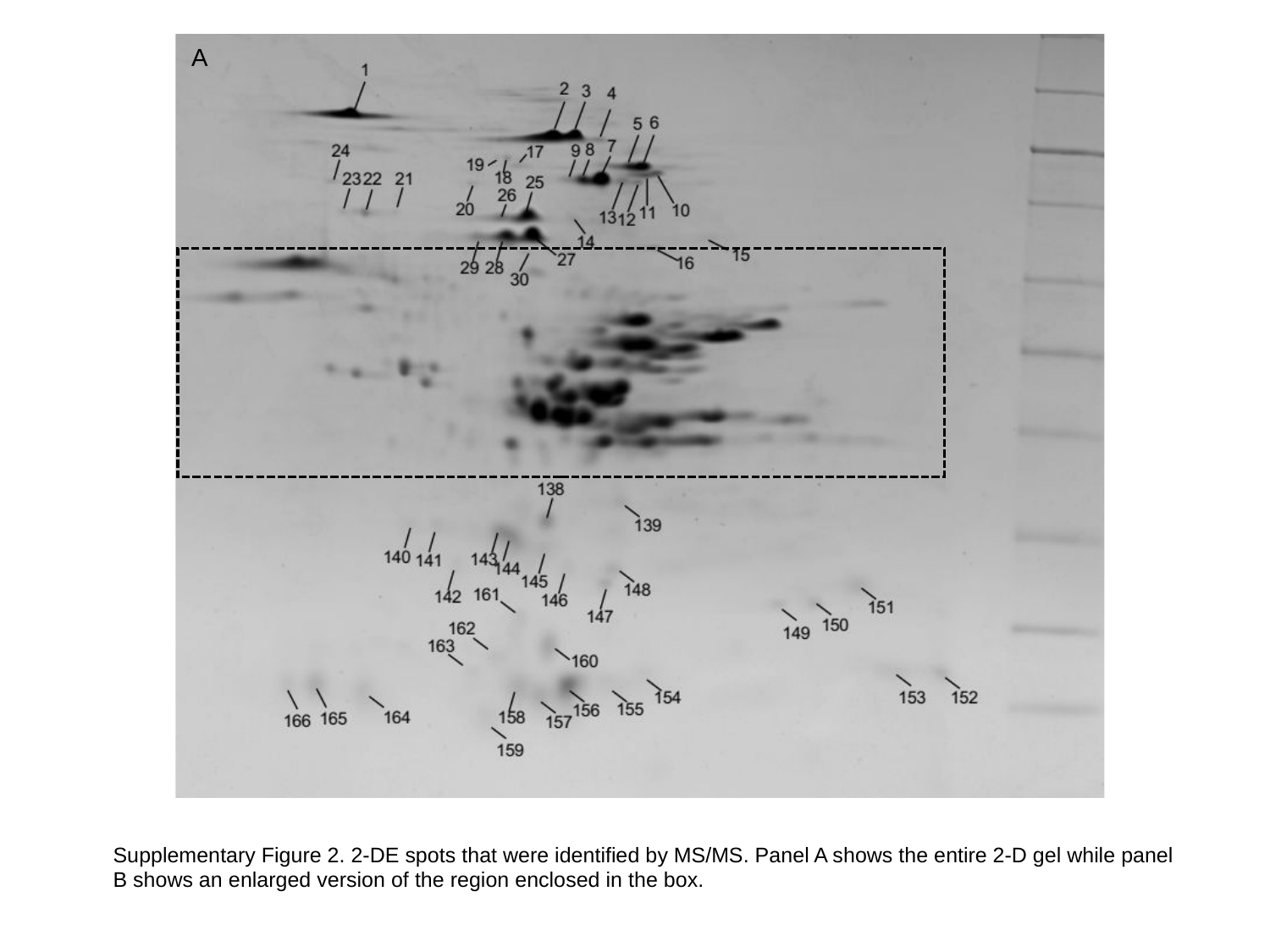

A
Supplementary Figure 2. 2-DE spots that were identified by MS/MS. Panel A shows the entire 2-D gel while panel B shows an enlarged version of the region enclosed in the box.

## Slide 2
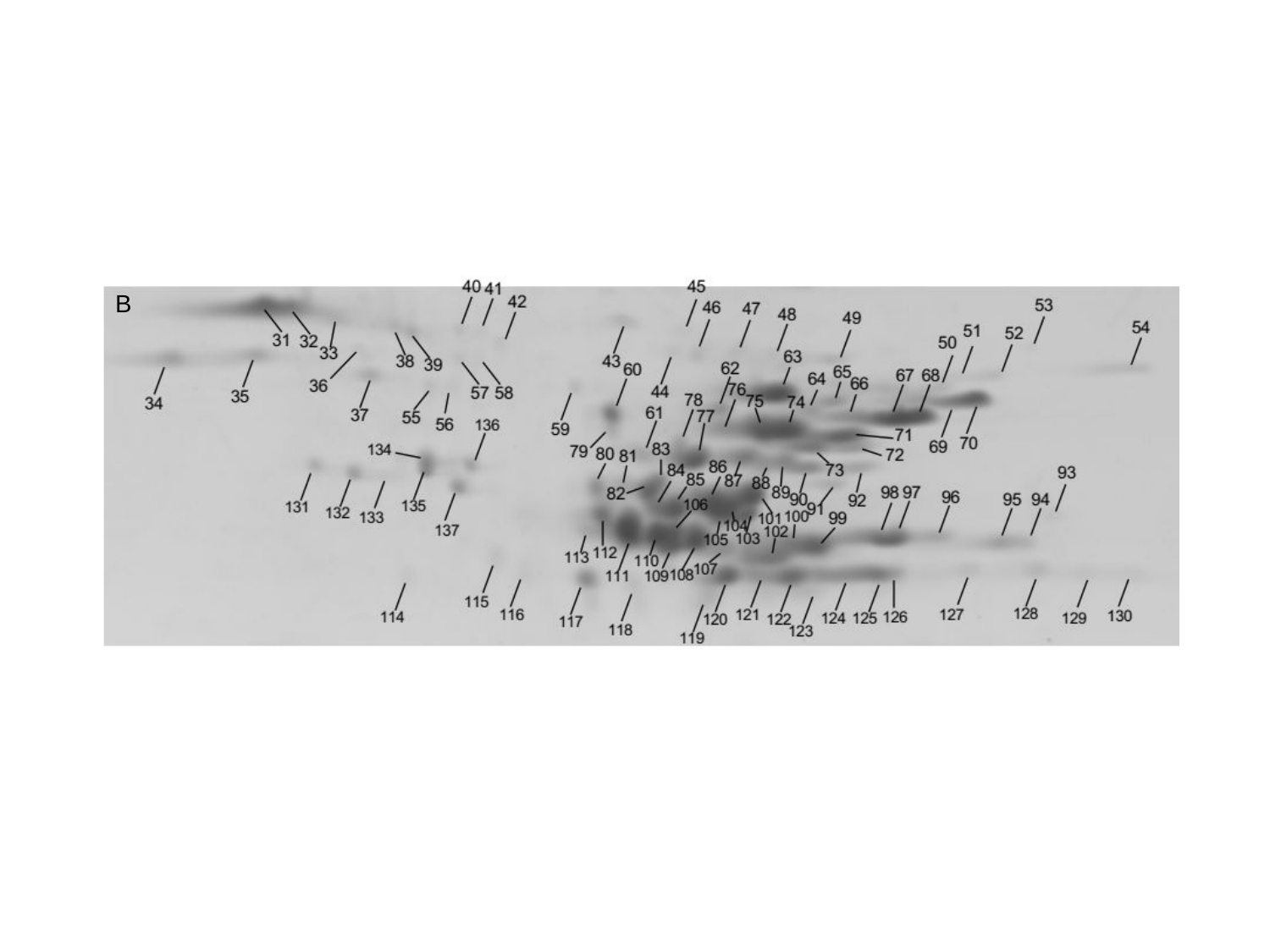

B
