# Supplementary material for: Exploiting the reference genome sequence of hexaploid wheat: a proteomic study of flour proteins from the cultivar Chinese Spring
Source: Funct Integr Genomics. 2019 Jun 27;20(1):1–16. doi: 10.1007/s10142-019-00694-z (PMC6954139; doi:10.1007/s10142-019-00694-z)
Supplement: Supplementary file 3 — Sequence comparison of two S-type LMW-GS showing a poly Q region that distinguishes the two proteins in the red box. (PPTX 53 kb) [file 10142_2019_694_MOESM3_ESM.pptx]

## Slide 1
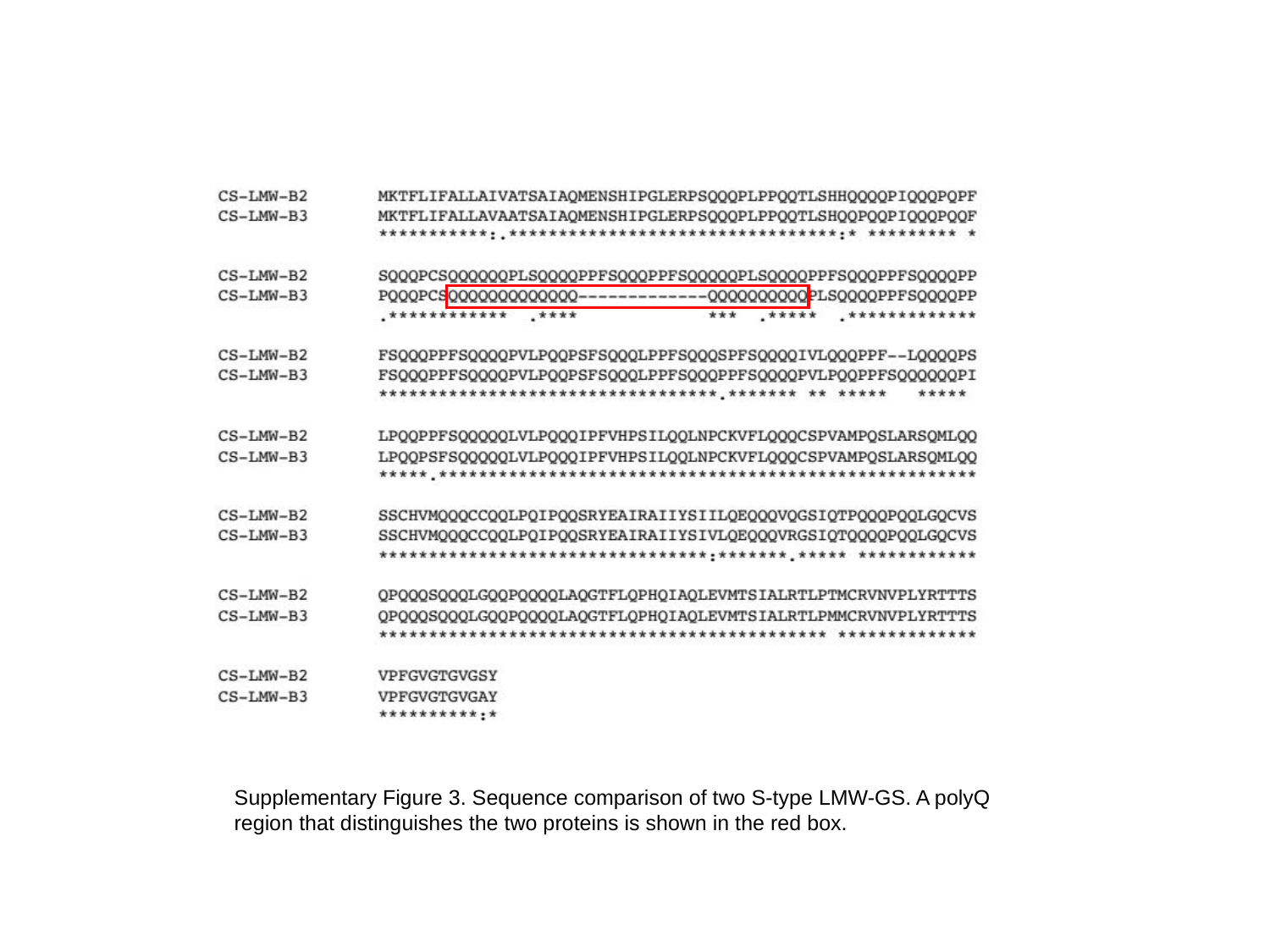

Supplementary Figure 3. Sequence comparison of two S-type LMW-GS. A polyQ region that distinguishes the two proteins is shown in the red box.
